# Endoscopic diagnosis and management of superficial esophageal squamous cell carcinoma

**DOI:** 10.1590/1806-9282.2024S133

**Published:** 2024-06-07

**Authors:** Renata Nobre Moura, Fauze Maluf-Filho

**Affiliations:** 1 Universidade de São Paulo, Cancer Institute of the State of São Paulo, São Paulo Medical School, Department of Gastroenterology – São Paulo (SP), Brazil.

## INTRODUCTION

**Figure 1:**
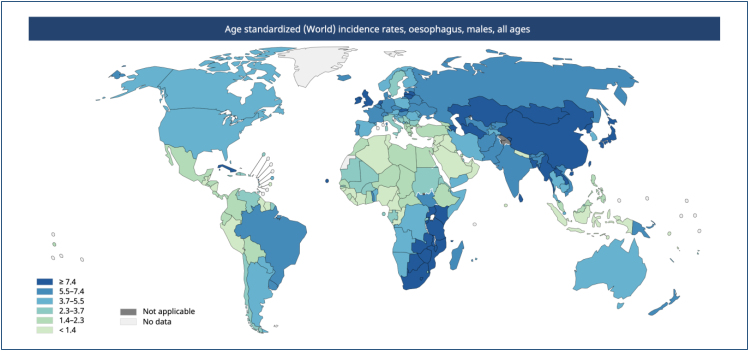
Incidence of esophageal cancer worldwide. Data source: GLOBOCAN 2020. Graph production: IARC (https://gco.iarc.fr/today) World Health Organization.

**Figure 2:**
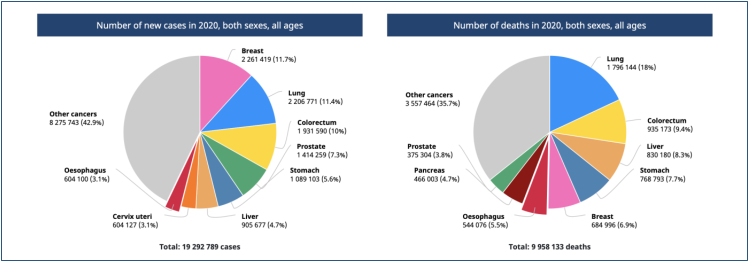
Number of new cases and deaths. Data source: GLOBOCAN 2020. Graph production: IARC (https://gco.iarc.fr/today) World Health Organization.

Esophageal neoplasia ranks seventh in incidence and sixth in mortality among all cancers worldwide^1^. Regarding histopathology, squamous cell carcinoma (SCC) accounts for **up to 90%** of cases and its distribution varies geographically, with a concentration in areas of greatest risk known as the **"esophageal cancer belt,"** which encompasses the region from northeast Iran, Central Asia, and northeast China^2^ (Figures 1 and 2).

Smoking and alcohol consumption are major risk factors for esophageal squamous cell carcinoma (ESCC). Patients with head and neck squamous cell carcinoma (HNSCC) are at risk of developing a second primary tumor on the esophagus supporting the concept of field cancerization. Results of a screening program in high-risk patients showed that the frequency of a second primary tumor in this population occurred in 8% of patients with HNSCC, mostly superficial lesions amenable to endoscopic curative resection. In multivariate analysis, SCC of the oral cavity and oropharynx and the presence of esophageal low-grade dysplasia (LGD) were the **predictive factors of ESCC** ^3^.

**Figure 3:**
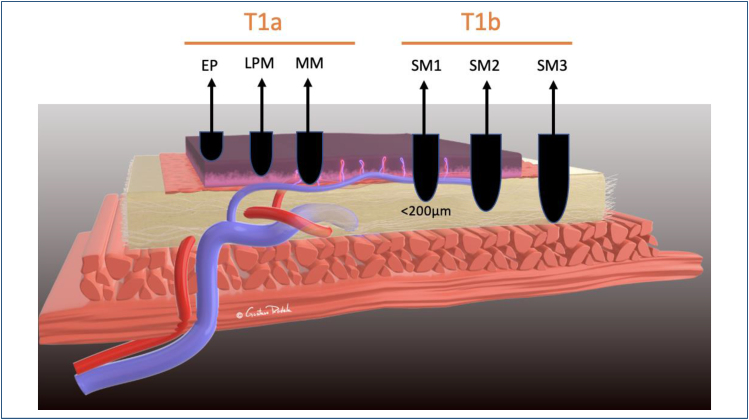
Subclassification for superficial cancer.

Survival rates and choice of initial treatment are directly related to invasion depth. According to the Japanese Esophageal Society^4^, superficial ESCC is defined as a cancer invading up to the submucosa, regardless of linfonodal invasion **(T1NxMx)**. On the contrary, early ESCC is the mucosal cancer **(T1aNxMx)** (Figure 3).

Management of ESCC has changed over the last few years, and endoscopic resection (ER) techniques have become increasingly important. Nevertheless, surgery continues to be the standard treatment, either alone or in combination with chemoradiotherapy. In addition to the tumor staging, the management of ESCC should be chosen according to patients’ preferences and the availability of surgical and endoscopic approaches.

As the incidence of ESCC is increasing mainly because of improvements in endoscopic detection, this review will focus on the advances in diagnosis and endoscopic treatment strategies for superficial ESCC.

## PRETREATMENT ASSESSMENT

### ENDOSCOPY

Most patients with superficial ESCC do not have signs or symptoms caused by the neoplasia. It means that the diagnosis of superficial ESCC relies on endoscopy mostly indicated for unrelated gastrointestinal symptoms (e.g., dyspepsia) or in the context of screening programs^5^.

**Figure 4:**
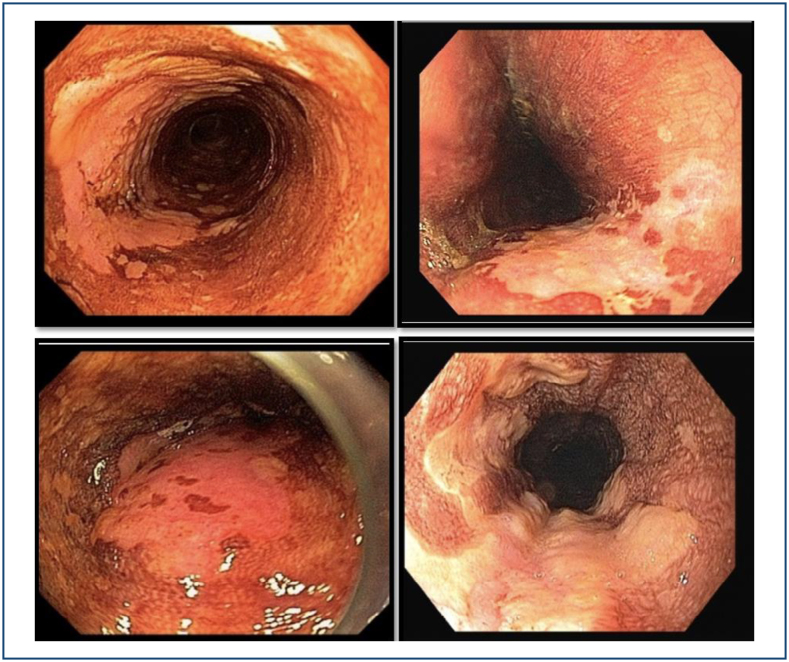
Lugol pink-color sign.

The accurate evaluation of disease extent is crucial for the selection of the appropriate treatment strategy, and the endoscopic assessment of tumor depth is essential. Nevertheless, **mucosal changes associated with early cancers may be subtle and missed**. Therefore, the right preparation for an endoscopic examination is mandatory. The first step is to remove mucus and bubbles from the mucosal surface with mucolytics and/or defoaming agents. Adequate conscious sedation is indicated. To avoid missing a lesion, it is essential to take time to evaluate the esophagus. It is estimated that high-definition, white light endoscopy (HD-WLE) has a **50% sensitivity** for the detection of ESCC. In this sense, Lugol chromo endoscopy was developed in the early 1990s. The principle is that iodine binds reversibly to glycogen, which is less abundant in immature and rapidly dividing cells such as those found in dysplasia and inflammation. Widely available today, Lugol's staining turned into an invaluable tool in characterizing the esophageal epithelial surface as a simple and cheap technique that improves the detection rate and helps to delineate margins. Compared with WLE, **Lugol's iodine chromoendoscopy significantly improved the sensitivity of ESCC**. However, this method has some drawbacks, namely, the lower specificity due to the non-differentiation of inflammatory changes and side effects such as chest pain^5-8^. A color change after iodine staining, from the initial yellow color to a pink color 2–3 min later, is known as the **pink-color sign** and is recognized as a valuable indicator for the diagnosis of ESCC^9,10^ (Figure 4). This sign has been reported to dramatically improve specificity for HGIN and invasive cancer. Compared with HD-WLE, electronic and optic chromoendoscopy (i.e., NBI, BLI, FICE, and i-scan) have a higher sensitivity for the diagnosis of ESCC. However, Lugol chromoendoscopy has still a higher sensitivity for this purpose.

**Figure 5:**
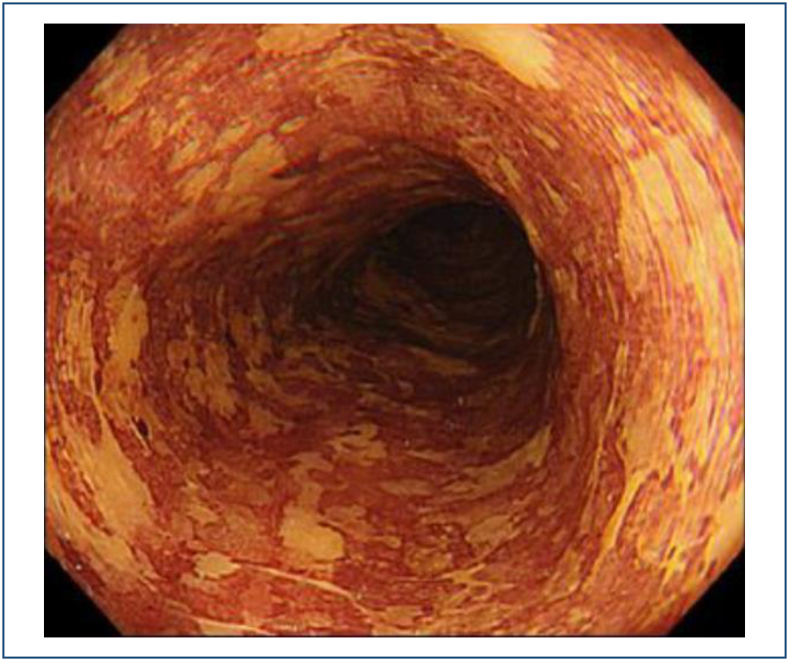
Leopard print pattern.

Because of its high specificity, the pink-color sign is a good indicator for choosing adequate biopsy sites in patients with multiple Lugol-void lesions (LVL), the so-called leopard print pattern (Figure 5).

The presence of multiple LVLs can indicate a high-risk condition for HGD and ESCC. Thus, the presence of multiple LVLs is important in clinical settings to assess the risk of development of ESCC^11^.

**Figure 6:**
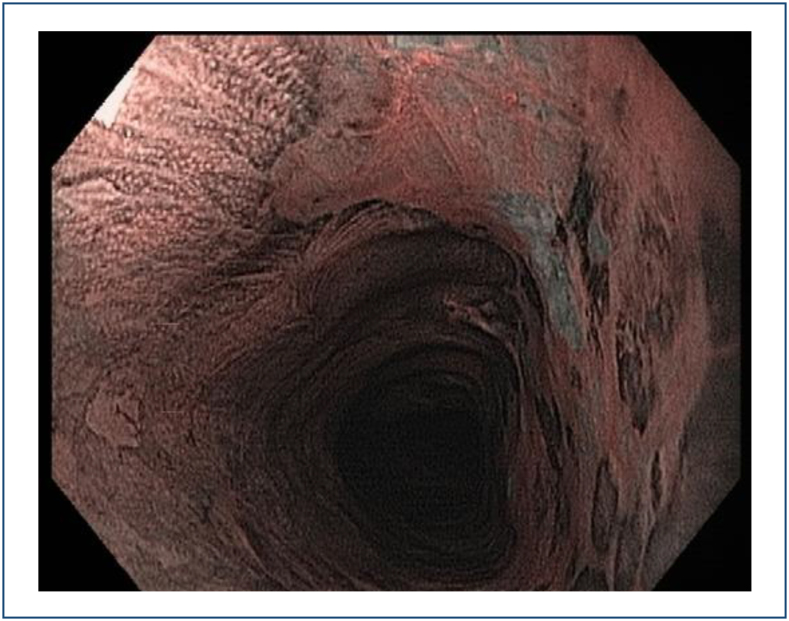
Metallic silver sign.

The pink-color sign is sometimes difficult to see because of its low intensity, whereas the metallic silver sign is clearly apparent with NBI. Its presence alone could indicate the presence of a cancerous lesion, regardless of macroscopic appearance or histopathologic characteristics^12^ (Figure 6).

**Figure 7:**
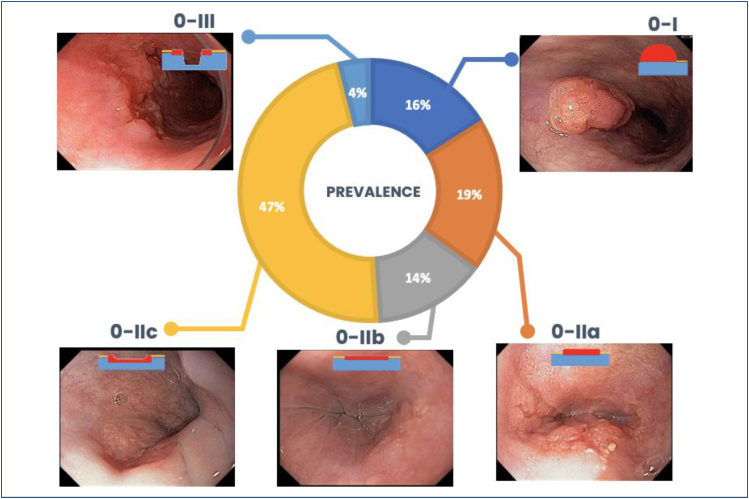
Macroscopic classification (the Paris Classification) and prevalence.

**Figure 8:**
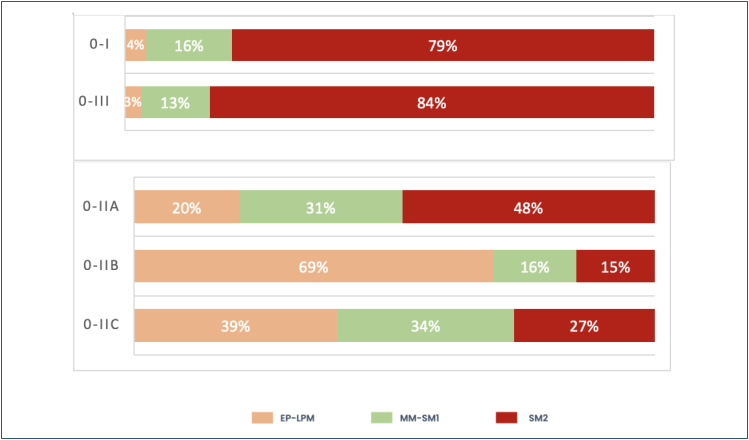
Invasion depth according to the Paris classification.

With HD-WLE, the macroscopic classification of Paris^13^ may help predict the extent of invasion into the submucosa. Polypoid and excavated lesions, classified as Paris Ip and III, respectively, are easy to recognize, but they account for only 20% of early cancer and are more likely to contain invasive submucosal cancer in more than 80% of the cases. By contrast, most early esophageal cancer has a flat appearance with minimal impact on the contour of the mucosal surface (0-IIa, IIb, and IIc) (Figures 7 and 8).

**Figure 9:**
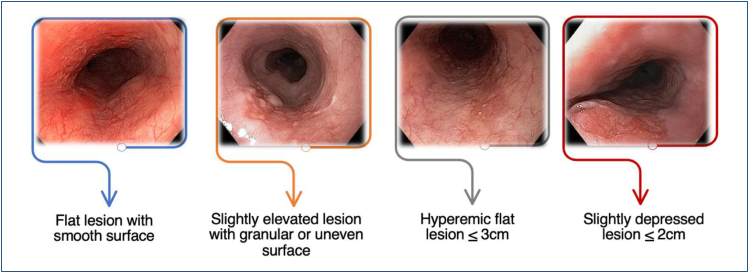
Macroscopic features of mucosal ESCC under HD-WLE.

**Figure 10:**
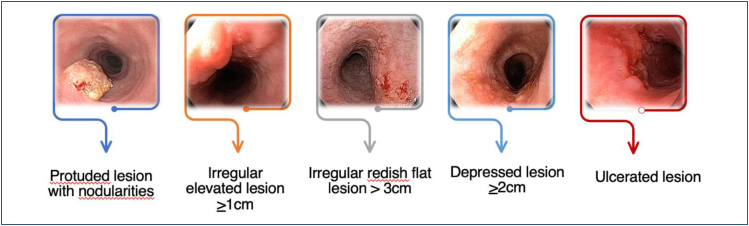
Macroscopic features of submucosal ESCC under HD-WLE.

Other macroscopic features of mucosal ESCC by HD-WLE are flat reddish areas with a smooth surface, slightly elevated or small depressed lesions with a slightly rough surface, or white granules (Figure 9). Submucosal ESCC may appear as irregular, protruded, and ulcerated lesions^14^ (Figure 10).

However, the sensitivity of the Paris classification for the prediction of the depth of invasion is only 50% even among experienced endoscopists. Therefore, endoscopic diagnosis based solely on this gross, macroscopic appearance of a tumor is of limited value. It is essential, therefore, to have an additional, more accurate staging method.

**Figure 11:**
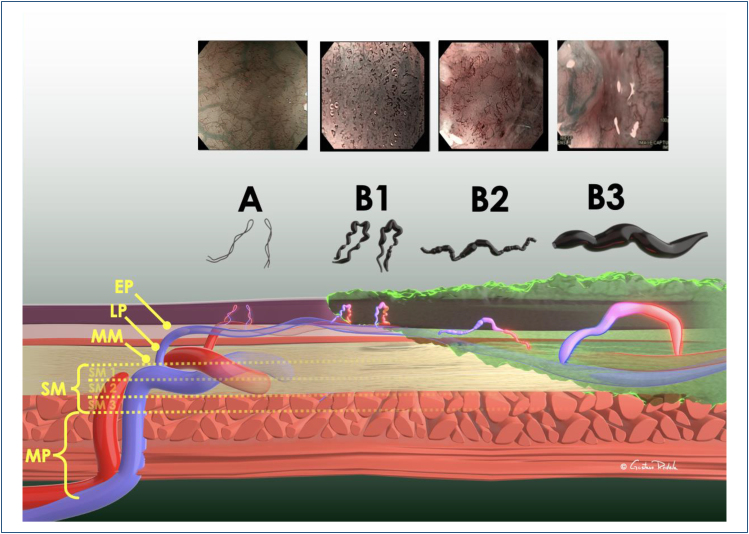
JES classification.

Magnifying endoscopic assessment of the intrapapillary capillary loops (IPCLs) can predict the depth of invasion^15,16^. In ESCC, IPCL pattern changes present as dilatation, weaving, change in caliber, and variety in shape, the so-called **"four characteristic markers of cancer."** According to the Japanese Esophageal Society classification^17^, microvessels are classified as type A if they have three or fewer factors and type B if they have all four. In this classification, vessels are classified into two categories: non-cancerous (normal epithelium, inflammation, and LGD) and cancerous (HGD and invasive SCC) epithelium. Type B1 is defined as type B vessels with a loop-like formation. B1 vessels normally appear as dot-like microvessels in a target area (Figure 11). When target lesions have only type B1 vessels, the histological invasion depth is predicted as T1a-EP (M1) or T1a-LPM (M2). B2 is defined as type B vessels without a loop-like formation that has a stretched and markedly elongated transformation. The B2 vessels often show a multilayered arrangement or irregularly branched/running pattern. This pattern is related to lesions invading muscularis mucosa (M3) and superficial submucosa (SM1, up to 200 micra). B3 is defined as highly dilated abnormal vessels whose caliber appears to be more than three times that of the usual B2 vessels and often appears green in color. The predicted invasion depth of the B3 pattern is deep submucosa.

## ENDOSCOPIC ULTRASOUND

**Figure 12:**
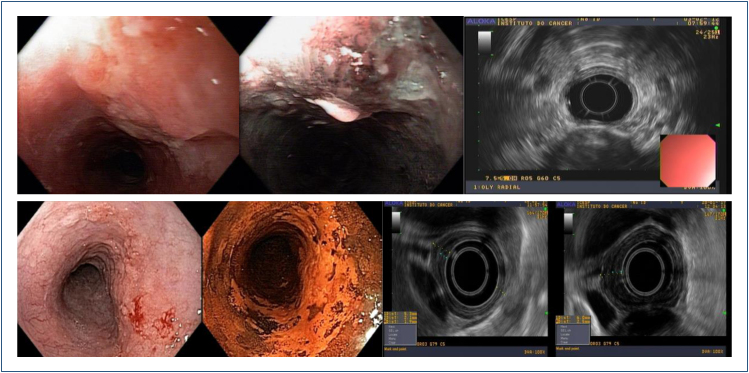
EUS assessment of T staging.

For locoregional staging of esophageal cancer of ESCC, endoscopic ultrasound (EUS) was extensively studied. It can be used for tumor (T) and node (N) staging (Figure 12). In general, EUS sensitivity and specificity rates for the correct evaluation of the T stage are 81–92% and 94–97%, respectively^18^. The overall accuracy for N staging is 74% when used alone^19^.

The usefulness of EUS in superficial cancer is controversial. An early meta-analysis of 19 studies and 1,019 patients with superficial esophageal cancer described an overall accuracy of 0.93 of EUS for T staging. However, the heterogeneity of this meta-analysis was high probably due to multiple factors including the location and type of lesion, the method and frequency of the EUS probe, and the experience of the endosonographer^20^. In our experience, **the EUS accuracy to differentiate T1a from T1b lesions is suboptimal** and we give preference to magnifying endoscopy. We indicate EUS in superficial ESCC when the findings of magnifying endoscopy are unclear aiming at a better T and N staging.

Moreover, in stenotic advanced tumors, EUS evaluation may not be technically possible. In a multicenter study involving 100 patients with stenotic esophageal neoplasms, the EUS scope could not traverse the stricture in 70. From them, all patients had T3Nx or T4Nx disease. This fact reduced the enthusiasm for tumor dilation to perform a complete EUS staging^21^.

## CROSS-SECTIONAL STUDIES

The evaluation for distant metastasis includes commonly computed tomography (CT) and/or positron emission tomography (PET-CT). These methods can also provide complementary information for T and N staging. Most superficial ESCCs are not detected on CT or PET-CT^22^.

## TREATMENT STRATEGY

**Figure 13:**
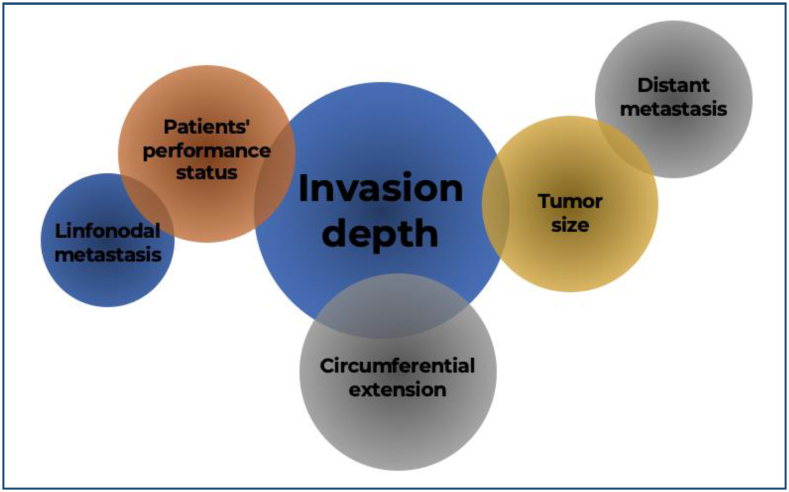
Therapeutic strategy for superficial esophageal squamous cell cancer.

The initial treatment strategy should take into consideration a multidisciplinary assessment of the patient's condition and choice, disease extension, metastatic status, invasion depth, tumor size, location, and circumferential extent (Figure 13).

**Figure 14:**
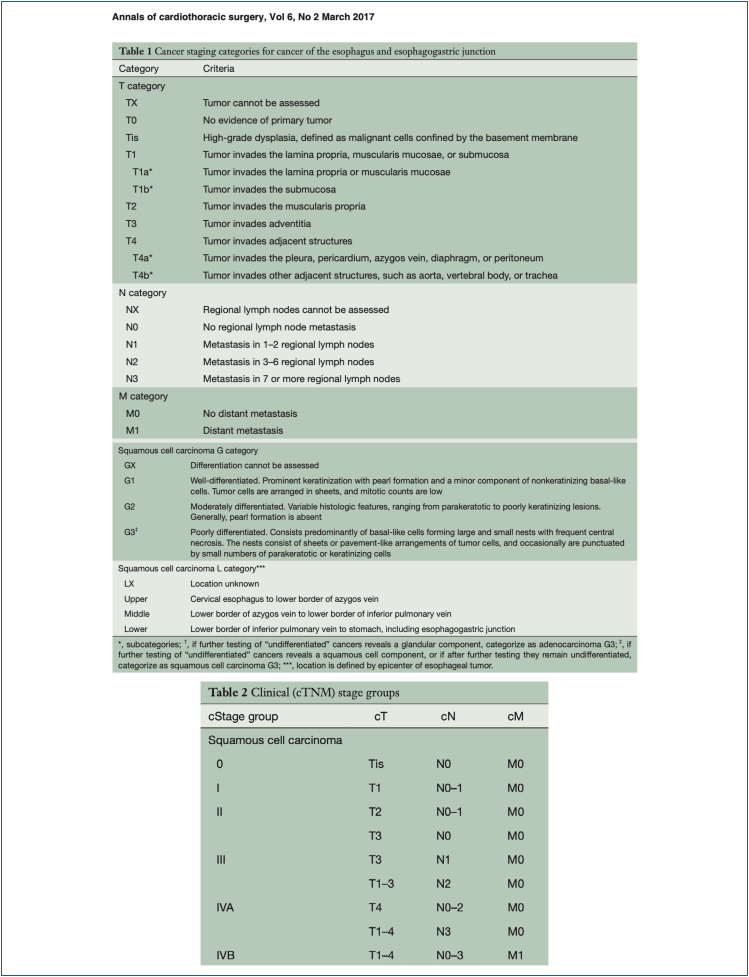
TNM stage according to the AJCC 8th edition. Available in Annals of Cardiothoracic Surgery, Vol. 6, No. 2, March 2017.

Among these factors, cancer invasion depth correlates with the risk of metastasis and curability. A proposed algorithm for the treatment based on the TNM stage (according to the AJCC 8th edition) is discussed below^23^ (Figure 14).

T1 (superficial) lesions are defined as those invading the mucosa (T1a) and submucosa (T1b). These lesions have been further categorized into three subtypes (M1–M3 and SM1–SM3, respectively) according to the depth of invasion.

**Figure 15:**
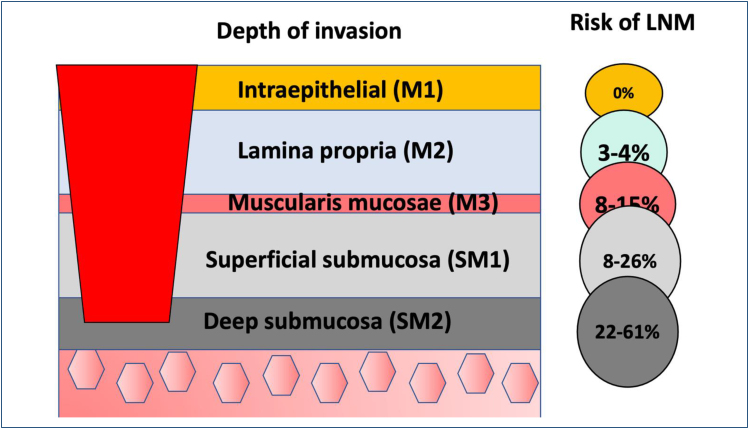
The correlation between superficial ESCC depth of invasion and the risk of lymph node metastasis.

Esophageal lesions classified as M1 (intraepithelial) or M2 (invades the lamina propria) have virtually no risk of lymph node involvement. This risk increases to 8–18% in lesions that invade the muscularis mucosa (M3), to 11–53% in lesions that invade the submucosa up to 200 μm (SM1), and 30–54% in deeper lesions (SM2)^17^. Additional characteristics that impact the risk of nodal involvement include vascular invasion, tumor size, and the degree of tumor differentiation (Figure 15).

Given the low risk of lymph node involvement, mucosal lesions classified as M1 and M2 (IPCL type B1) are absolute indications for ER. Lesions clinically classified as invading muscularis mucosa (M3) or superficial submucosa (SM1) can also be treated by ER. However, due to the risk of linfonodal metastasis, they are considered relative indications. Lesions with endoscopic features of deep submucosa invasion (more than 200 μm or ≥SM2) are associated with a risk of lymph node metastasis at a frequency of about 50% and should be treated similarly to advanced carcinomas^24-27^.

**Figure 16:**
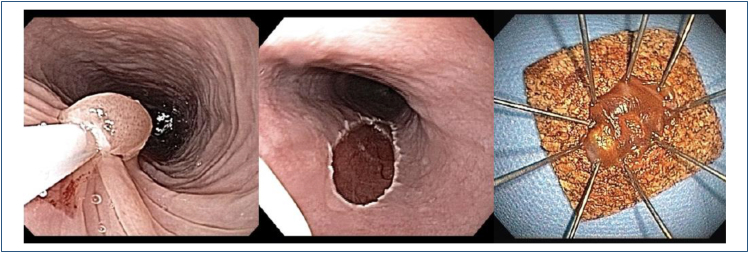
Esophageal endoscopic mucosal resection (EMR).

**Figure 17:**
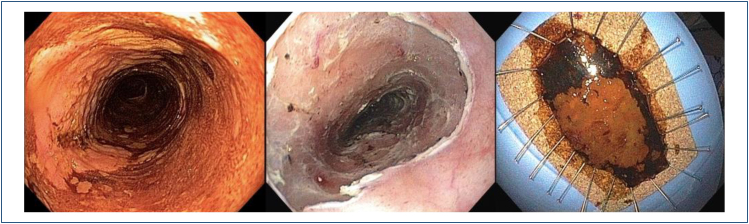
Endoscopic submucosal dissection (ESD).

**Figure 18:**
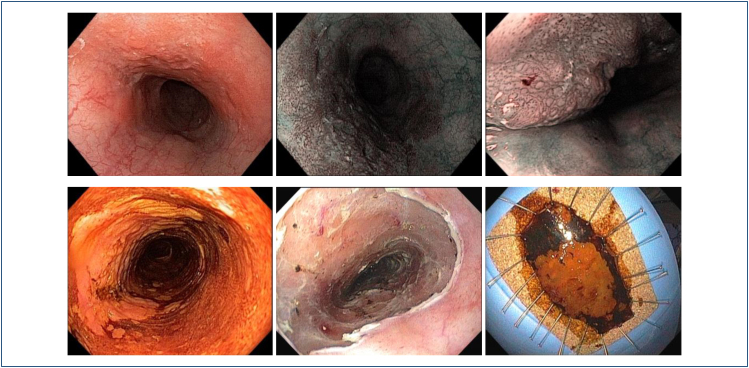
Circumferential ESD.

Endoscopic techniques have been developed for curative resection of superficial neoplasms of the esophagus, such as endoscopic mucosal resection (EMR, Figure 16) and endoscopic submucosal dissection (ESD, Figure 17). Currently, **ESD is considered the preferred approach to manage superficial ESCC, enabling accurate** *en bloc* **resection with a lower recurrence rate and improved survival (Figure 18)** ^28-31^.

In a multicenter retrospective study that included 148 tumors (80 treated by EMR and 68 by ESD), the recurrence rate was significantly higher in the EMR group (23.7 versus 2.9%), and 5-year recurrence-free survival rates were worse (73.4 versus 95.2%)^3,32^ in the EMR group.

In comparison with surgery, even though no randomized trials are available, evidence shows that the long-term outcomes of ESD and surgery are comparable. In a retrospective study, 116 T1a ESCCs larger than 2 cm treated either surgically (n=47) or endoscopically (n=69) were compared. The overall survival rate was similar (97.1% versus 91.5%, p=0.18), Procedure-related complications occurred more often in the surgical group (8.5% versus 0, p<0.05)^33^.

In addition to the depth of invasion, the circumferential extent of the lesion should be taken into consideration because of the high risk of stenosis in lesions involving more than 75% of the circumference. Nevertheless, more effective prophylaxis with oral and/or intravenous corticosteroids has recently been developed with promising results^34,35^. Furthermore, dilatation is another effective method to prevent stenosis following post-ESD stenosis. In terms of outcomes, the complete resection rate following circumferential esophageal ESD is reported to be as high as 100% and the curative resection rate is 70%^36-38^.

**Figure 19:**
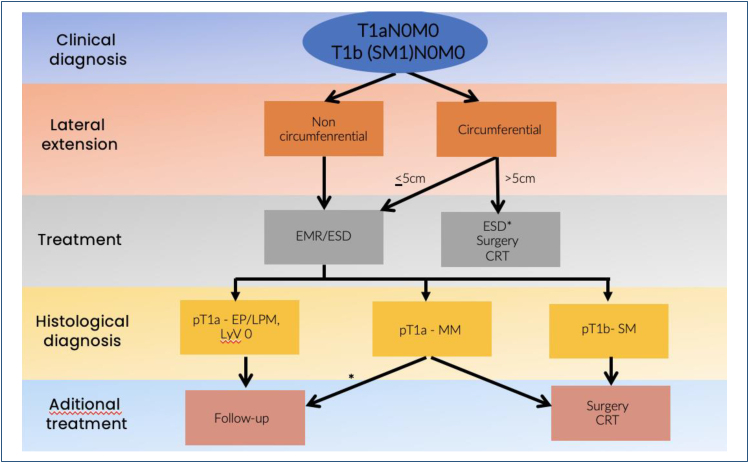
Therapeutic strategy for superficial ESCC. Adapted from Ishiara et al. Dig Endosc, 2020.

It is important to highlight that the endoscopic diagnosis of the invasion depth has some limitations, mostly on extensive lesions and lesions with IPCL Type B2, where the JES classification accuracy is only 55.7%^26^. Accordingly, the assessment of the histological diagnosis of resected specimens is essential. In patients classified as having pT1a-epithelium/lamina propria mucosae disease (M1 or M2), follow-up should be scheduled. On the contrary, in patients with muscularis mucosa (M3) or superficial submucosa (SM1) and positive vascular invasion, an additional treatment (surgical or chemoradiotherapy) is required. Also, for lesions showing deep submucosal invasion, regardless of lymphovascular metastasis, additional esophagectomy or chemoradiotherapy is necessary^27^. The selection between surgery and chemoradiotherapy should be made after assessing the patient's clinical condition (Figure 19).

A Japanese trial^39^ evaluated the efficacy of ER followed by chemoradiotherapy. Patients with histologically M3 lesions, positive vascular invasion, and negative resection margins or histologically SM invasion and negative resection margin underwent prophylactic chemoradiotherapy. Patients with SM invasion and positive resection margin underwent definitive chemoradiotherapy. Favorable results were obtained in the prophylactic chemoradiotherapy group, with a 3-year overall survival rate of 90.7% (90%CI 84.0–94.7%). That study showed that even when ER is not curative, a good prognosis can be expected if additional chemoradiotherapy is administered.

A multicenter study involving seven western centers reported a 25% residual/recurrence rate of esophageal cancer (both adenocarcinoma and ESCC) after ESD for T1b lesions (hazard ratio, 6.25; 95% confidence interval, 1.29–30.36; p=0.023). Those findings corroborate the limitation of ER for esophageal cancer with submucosa invasion^40^.

## CONCLUSION

Superficial ESCC diagnosis has been increasing worldwide. The endoscopic prediction of the depth of tumor invasion is the most important factor in selecting the treatment strategy and optimizing outcomes. ER techniques by EMR and ESD have become the most important treatment as provide high curative rates and organ preservation.
